# Etiological serotype and genotype distributions and clinical characteristics of group B *streptococcus*-inducing invasive disease among infants in South China

**DOI:** 10.1186/s12887-020-02048-2

**Published:** 2020-04-02

**Authors:** Yao Zhu, Jiayin Wu, Xinyi Zheng, Dengli Liu, Liping Xu, Dongmei Chen, Wenying Qiu, Zhongling Huang, Ronghua Zhong, Ling Chen, Mingyuan He, Simin Ma, Yayin Lin, Xinzhu Lin, Chao Chen

**Affiliations:** 1 Department of Neonatology, Women and Children’s Hospital of Xiamen University, Xiamen, 361003 China; 2 Department of Clinical Laboratory, Women and Children’s Hospital of Xiamen University, Xiamen, China; 3 School of Public Health of Xiamen University, Xiamen, China; 4 Department of Neonatology, The First Affiliated Hospital of Xiamen University, Xiamen, China; 5 Department of Neonatology, Zhangzhou Affiliated Hospital of Fujian Medical University, Zhangzhou, China; 6 Department of Neonatology, Quanzhou Women and Children’s Hospital, Quanzhou, China; 7 Department of Neonatology, Longhai First Affiliated Hospital, Longhai, China; 8 Department of Neonatology, Zhangzhou Zhengxing Hospital, Zhangzhou, China; 9 Department of Neonatology, Longyan First Affiliated Hospital of Fujian Medical University, Longyan, China; 10 Department of Neonatology, Children’s Hospital of Fudan University, Shanghai, China

**Keywords:** Group B *streptococcus*, Serotype, MLST, Newborn infant, GBS-EOD, GBS-LOD

## Abstract

**Background:**

Group B *streptococcus* (GBS)-induced invasive disease is a major cause of illness and death among infants aged under 90 days in China; however, invasive GBS infection remains unknown in China. We aimed to describe the serotype and genotype distributions of early-onset disease (EOD) and late-onset disease (LOD), and to show the clinical correlations among various GBS serotypes and genotypes obtained from infants with invasive GBS infections.

**Methods:**

Between June 1, 2016 and June 1, 2018, 84 GBS strains were collected from patients younger than 90 days at seven Chinese hospitals. Clinical data were retrospectively reviewed. GBS serotyping was conducted and multi-locus sequence typing was performed.

**Results:**

Serotypes Ia, Ib, II, III, and V were detected. Serotype III (60.71%) was the most common, followed by Ia (16.67%) and Ib (14.29%). Intrapartum temperature ≥ 37.5 °C, chorioamnionitis, and mortality were noted in 28.57, 42.86, and 28.57% of patients with serotype Ia, respectively, and these rates were higher than those in patients with serotypes Ib and III (*P* = 0.041, *P* = 0.031, and *P* = 0.023, respectively). The incidence of respiratory distress was lower (*P* = 0.039) while that of purulent meningitis was higher (*P* = 0.026) in the serotype III group. Eighteen sequence types were detected among isolates, and ST17 [42.86% (36/84)] was the most prevalent.

**Conclusions:**

GBS isolates belonging to serotypes Ia, Ib, and III are common in southern mainland China, and ST17 is highly prevalent. Differences were found in the clinical manifestations of invasive GBS disease induced by serotypes Ia and III.

## Background

Group B *streptococcus* (GBS), also referred to as *Streptococcus agalactiae*, is the sole member of the Lancefield group and a major cause of invasive infections in infants, especially those living in China, due to the lack of routine screening for GBS maternal colonization and intrapartum antibiotic prophylaxis (IAP) implementation. GBS is one of the main pathogens responsible for morbidity and mortality among infants in many countries, including China [1, 2]. The incidence of invasive GBS infection among newborns and infants varies greatly around the world, from 0.57/1000 live births in Europe to 1.21/1000 live births in Africa [3]. There is a paucity of data on the prevalence of invasive GBS infections in infants in China. Further, there are speculations that China may loosen the current two-child limit; thus, leading to the birth of more infants every year. Hence, it is very important to reduce the rate of these infections among infants in China.

The distribution of serotypes is closely related to the epidemiology of GBS infections. On the basis of the composition of capsular polysaccharide (CPS), the following ten serotypes are currently recognized: Ia, Ib, and II-IX [4, 5]. The prevalence of different serotypes varies according to the time and geographic origin. Five studies in middle-income countries showed that serotype III accounted for nearly half of the isolates, followed by serotypes Ia, II, and V [3]. The most prevalent serotypes (Ia, Ib, II, III, and V) have been reported to account for over 96% of serotypes in the United States, 93% in Europe, and 89% in the Western Pacific [6]. However, the distribution of GBS serotypes in Asia has been sparsely surveyed.

In young infants, invasive GBS infections are usually categorized into early-onset disease (EOD, occurring at the age of 0–6 days) and late-onset disease (LOD, occurring at the age of 7–89 days).

In the United States, IAP has decreased the incidence of GBS-EOD from 1.7 cases per 1000 live births in 1993 to 0.4 cases per 1000 live births in 2008 [7]. However, IAP cannot prevent GBS-LOD [8]. In addition, widespread IAP can cause anaphylaxis or lead to the development of antibiotic-resistant strains. An epidemiological study showed that the distribution of serotypes in a pathogen is an important precondition for formulating serotype-based vaccines [9]. A safe and efficacious vaccine against the most common serotypes can prevent most infant GBS infections (early and late onset infections).

Thus, our study is aimed at providing new information on GBS serotypes and the associated clinical features of invasive GBS isolates in a Chinese population and obtaining reference values for developing methods to prevent GBS infections.

## Methods

### Subject population

Seven hospitals reported 95,941 live births and an incidence of invasive GBS disease of 0.88 cases/1000 live births during the study period. Eighty-four GBS strains were obtained from the Women and Children’s Hospital of Xiamen University (Xiamen, China), The First Affiliated Hospital of Xiamen University (Xiamen, China), Zhangzhou Affiliated Hospital of Fujian Medical University (Zhangzhou, China), Longyan First Affiliated Hospital of Fujian Medical University (Longyan, China), Quanzhou Women and Children’s Hospital (Quanzhou, China), Longhai First Affiliated Hospital (Longhai, China), and Zhangzhou Zhengxing Hospital (Zhangzhou, China). Invasive GBS strains were collected prospectively from normally sterile sites (tracheal secretions, gastric fluid, blood, and cerebrospinal fluid). The detection of GBS strains was performed at the bacteriological laboratory of the Women and Children’s Hospital of Xiamen University between June 1, 2016 and June 1, 2018. GBS strains obtained from patients younger than 90 days and the medical records of these patients were retrospectively reviewed. A questionnaire was designed to collect clinical information, including age, symptoms, laboratory data, antibiotic usage, complications, length of hospital stay, gestational age, birth weight, and maternal history. The study protocol was in strict accordance with the ethical standards of the respective regional committee on human experimentation and the Helsinki Declaration of 1975 (revised in 1983). The ethics committee of Xiamen Maternal and Child Care Hospital of human body research approved the study (approval no. KY-2019-033), and the parents of all study participants provided written informed consent.

### Definitions

Invasive GBS disease was defined as the isolation of GBS from a normally sterile site using conventional microbiological methods along with signs of clinical disease, such as sepsis, pneumonia, or meningitis [10] GBS-EOD was defined as invasive GBS disease in newborns 0–6 days of age, and GBS-LOD was defined as invasive GBS disease in infants 7–89 days of age [10].

#### Serotyping

Bacteria were cultured in sheep blood agar plates and confirmed using the Christie, Atkins, and Munch Petersen (CAMP) test and a commercially available Streptococcal Grouping Kit according to the methods described in a previous paper [11]. After culture, all 84 strains were confirmed to be GBS and the isolates were serotyped using a latex agglutination kit (reagents Ia, Ib, and II–IX; Statens Serum Institut, Copenhagen, Denmark) [12].

### Multi-locus sequence typing (MLST)

Chromosomal DNA was extracted from overnight cultures of isolates cultivated at 35 °C on 5% Müeller-Hinton agar using a DNA Mini Kit (QIAGEN, Germany) according to the manufacturer’s instructions. Seven housekeeping genes (*adhP*, *pheS*, *atr*, *glnA*, *sdhA*, *glcK*, and *tkt*) were amplified with PCR using oligonucleotide primers [11]. The amplification products were sequenced by Shenzhen Huada Gene Technology Co. Ltd. The amplification and sequencing primers were submitted to the GBS MLST database (http://pubmlst.org/sagalactiae/info/primers.shtml) for the purpose of designation. We used the Chromas Lite software (version 2.6.5, Technelysium Pty. Ltd., Tewantin, Queensland, Australia) for correction and the MLST database (http://pubmlst.org/sagalactiae) to assign alleles at the seven loci. We defined each isolate by the sequence type (ST) [13].

### Statistical analysis

SPSS Statistics for Windows, Version 25.0 (IBM Corp., Armonk, NY, USA) was used to perform statistical analysis. The data for age and the length of hospital stay are presented as medians and interquartile ranges. Qualitative variables were compared using the Chi-square or Fisher’s Exact test. N**umerical** variables were compared using analysis of variance or non-parametric tests (Kruskal-Wallis *H* test). The Kaplan-Meier method was used for the analysis of survival time. A log-rank test was used to compare the survival curves among various serotypes. Differences were deemed statistically significant at *P* < 0.05.

## Results

### Serotype distribution

**Fig. 1:**
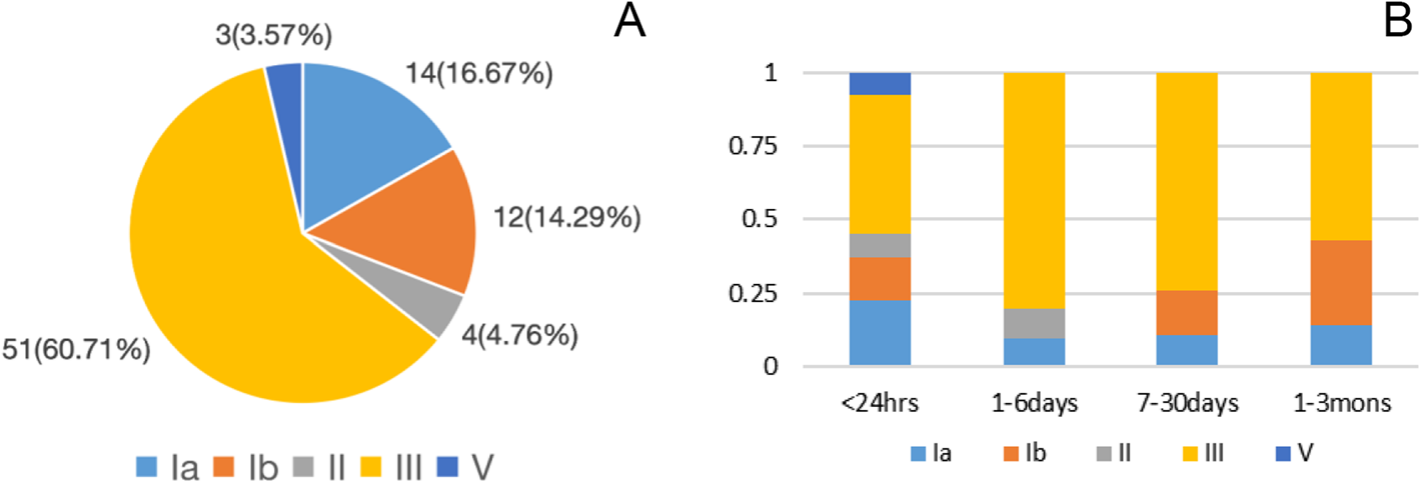
**a** Percentage of serotypes among 84 GBS isolates. Serotypes Ia, Ib, II, III, and V are indicated by individual colors. **b** Percentage of serotypes among different age groups. Serotypes Ia, Ib, II, III, and V are indicated by individual colors. < 24 h: Ia: 9 (22.50%), Ib: 6 (15.00%), II: 3 (7.50%), III: 19 (47.50%), V: 3 (7.50%); 1–6 days: Ia: 1 (10.00%), II: 1 (10.00%), III: 8 (80.00%); 7–30 days: Ia: 3 (11.11%), Ib: 4 (14.81%), III: 20 (74.08%); and 1–3 mons: Ia: 1 (14.29%), Ib: 2 (28.57%), III: 4 (57.14%)

In our study, five serotypes were detected among the 84 GBS isolates. The most prevalent serotype was III, accounting for 60.71% (51/84) of all isolates. This was followed by serotype Ia that accounted for 16.67% (14/84) of all isolates, Ib that accounted for 14.29% (12/84) of all isolates, II that accounted for 4.76% (4/84) of all isolates, and V that accounted for 3.57% (3/84) of all isolates. These findings are presented in Fig. 1 (a). Fifty newborns developed GBS-EOD and thirty-four infants developed GBS-LOD. There was no significant difference in the proportion of GBS serotypes between the two groups (all *P >* 0.05, Table 1).

**Table 1:** Distribution of GBS serotypes in 84 isolates belonging to the two different age groups

Groups	n	Ia	Ib	II	III	V
GBS-EOD	50	10 (20.00)	6 (12.00)	4 (8.00)	27 (54.00)	3 (6.00)
GBS-LOD	34	4 (11.76)	6 (17.65)	0 (0.00)	24 (70.59)	0 (0.00)
*χ*^2^ value		0.988	0.167	1.364	2.335	0.732
*P*		0.320	0.683	0.243	0.127	0.392

As shown in Fig. 1 (b), the serotype distribution varied at different ages of disease onset. Serotype III was the most prevalent in patients of all age groups. Serotype Ia was the second most prevalent in GBS-EOD patients (< 6 days), whereas serotype Ib was the second most prevalent in GBS-LOD patients (7 days - 3 months).

### Comparison of serotype III infections and non-type III infections

**Fig. 2:**
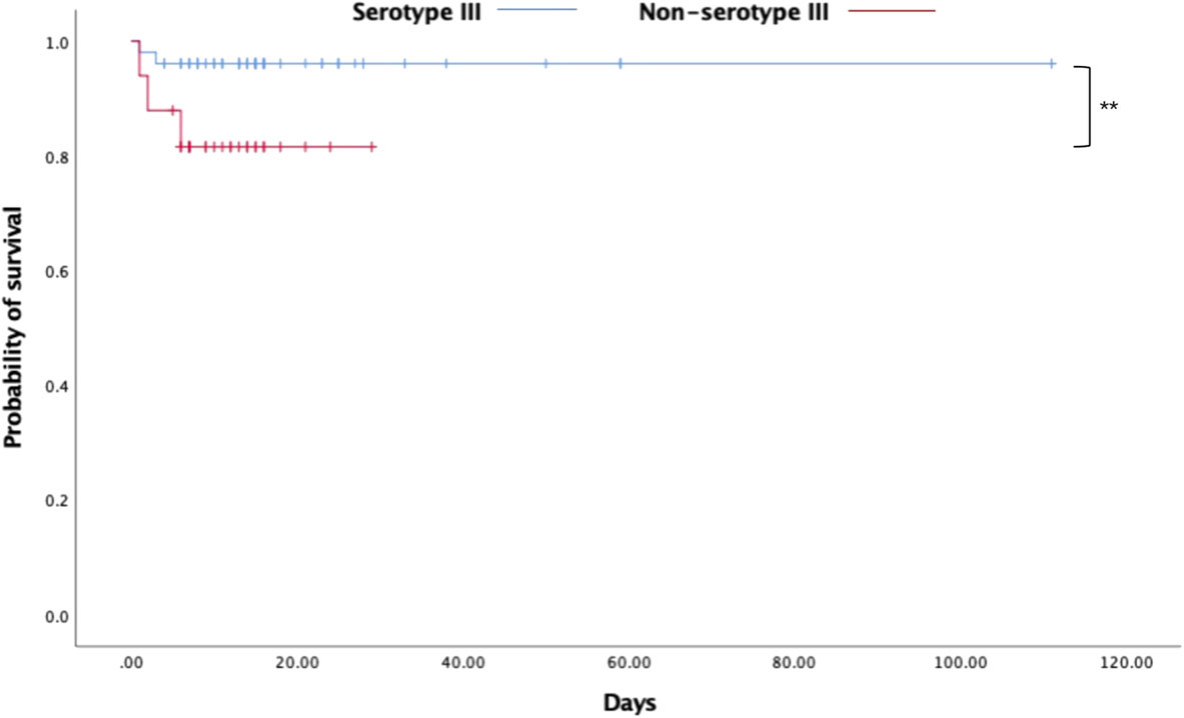
Kaplan-Meier plot survival curves categorized by serotype III (*n* = 51) and non-serotype III (*n* = 33). **p = 0.031

In our study, serotypes Ia, Ib, and III induced 91.67% of infections; therefore, the clinical parameters of infants in the three serotype groups were compared (Tables 2 and 3). Intrapartum temperature ≥ 37.5 °C and chorioamnionitis were noted in 28.57 and 42.86% of patients with serotype Ia, respectively, and these percentages were higher than those in patients with serotypes Ib and III (*P* = 0.041 and *P* = 0.031). There was a statistically significant difference in the rate of respiratory distress, purulent meningitis, and mortality among the three groups. The incidence of respiratory distress in the serotype III group (29.41%) was lower than that in the serotype Ia and Ib groups (*P* = 0.039). Purulent meningitis was noted in 41.18% of patients with serotype III, and this percentage was higher than that in patients with serotypes Ia and Ib (*P* = 0.026). Mortality in the serotype Ia group was 28.57%, which was markedly higher than that in the serotype III group (3.92%), and there was no case of death in the serotype Ib group (*P* = 0.023). The total mortality rate among infants in this study was 7.14% (6/84). The survival curve for infants with serotype III infections was significantly better than that for infants with non-type III infections (*P* = 0.031, Fig. 2).

**Table 2:** Demographics and maternal characteristics of infants with serotypes Ia, Ib, and III

Characteristics	Serotype Ia (*n* = 14)	Serotype Ib (*n* = 12)	Serotype III (*n* = 51)	F /*χ*^2^ value	*P*
Age, median (IQR), days	0.38 (0.04,15.25)	7.71 (0.20,23.00)	6.00 (0.17,18.00)	1.590	0.228
0–6 days, n (%)	10 (71.43)	6 (50.00)	27 (52.94)	1.719	0.423
7–89 days, n (%)	4 (28.57)	6 (50.00)	24 (47.06)		
Male gender, n (%)	8 (57.14)	6 (50.00)	29 (56.86)	0.197	0.906
Preterm (GA < 37 weeks), n (%)	2 (14.29)	2 (16.67)	9 (17.65)	0.092	0.955
Low birth weight (< 2500 g), n (%)	3 (21.43)	2 (16.67)	10 (19.61)	0.097	0.953
Small for gestation age, n (%)	3 (21.43)	0 (0.00)	6 (11.76)	4.049	0.132
< P10	3 (21.43)	0 (0.00)	3 (5.88)	3.764	0.115*
< P3	0 (0.00)	0 (0.00)	3 (5.88)	0.695	1.000*
Birth via cesarean section, n (%)	4 (28.57)	5 (41.67)	13 (25.49)	1.180	0.554
Regular antenatal screening, n (%)	12 (85.71)	11 (91.67)	48 (94.12)	1.567	0.565*
Amniotic membrane rupture ≥18 h, n (%)	3 (21.42)	1 (8.33)	9 (17.65)	0.956	0.620
Intrapartum temperature ≥ 37.5 °C, n (%)	4 (28.57)	1 (8.33)	2 (3.92)	6.403	0.041
MSAF^a^, n (%)	3 (21.43)	4 (33.33)	8 (15.69)	1.803	0.406
Chorioamnionitis^b^, n (%)	6 (42.86)	1 (8.33)	6 (11.76)	6.969	0.031
Gestational vaginitis, n (%)	4 (28.57)	2 (16.67)	13 (25.49)	0.580	0.748
Gestational bacteriuria, n (%)	1 (7.14)	0 (0.00)	2 (3.92)	1.003	0.715*
GBS disease in infants from previous pregnancies, n (%)	1 (7.14)	0 (0.00)	1 (1.96)	1.787	0.564*
GBS antenatal screening, n (%)					
Done	12 (85.71)	9 (75.00)	41 (80.39)	0.479	0.787
Not done	2 (14.29)	3 (25.00)	10 (19.61)		
Positive	9 (64.29)	6 (50.00)	28 (54.90)	0.589	0.745
Negative	3 (21.43)	3 (25.00)	13 (25.49)	0.101	0.951
Standard IAP, n (%)	5 (35.71)	4 (33.33)	20 (39.22)	0.172	0.917
Postpartum GBS mastitis, n (%)	0 (0.00)	1 (8.33)	2 (3.92)	1.311	0.471

^a^Meconium-stained amniotic fluid

^b^Chorioamnionitis (CAM): Clinical manifestations include intrapartum fever (temperature ≥ 38 °C) alone or concomitant with maternal leukocytosis, tenderness over the uterus, foul-smelling amniotic fluid, maternal and/or fetal tachycardia, and positive placental pathology

*Fisher’s exact test

**Table 3:** Clinical characteristics of infants with serotypes Ia, Ib, and III

Characteristics	Serotype Ia (*n* = 14)	Serotype Ib (*n* = 12)	Serotype III (*n* = 51)	F /*χ*^2^ value	*P*
**Laboratory data**					
WBC^a^, mean ± SD, 10^3^/μL	15.44 ± 7.78	23.13 ± 7.24	15.83 ± 8.26	2.747	0.071
Platelets, mean ± SD, 10^3^/μL	357.71 ± 257.28	437.25 ± 224.59	390.80 ± 218.06	0.401	0.671
Abnormal PCT^b^, n (%)	2 (14.29)	3 (25.00)	17 (33.33)	2.230	0.328
CRP^c^, mean ± SD, mg/L	32.20 ± 38.84	62.29 ± 75.50	54.84 ± 66.49	0.874	0.421
**Clinical feature**					
Fever presentation, n (%)	7 (50.00)	6 (50.00)	31 (60.78)	0.818	0.664
Respiratory distress^d^, n (%)	7 (50.00)	8 (66.67)	15 (29.41)	6.485	0.039
Seizure presentation, n (%)	1 (7.14)	1 (8.33)	4 (7.84)	0.341	1.000*
Pneumonia, n (%)	10 (71.43)	10 (83.33)	31 (60.78)	2.604	0.286
Sepsis, n (%)	8 (57.14)	6 (50.00)	37 (72.55)	2.777	0.249
Purulent meningitis, n (%)	1 (7.14)	3 (25.00)	21 (41.18)	7.268	0.026
Pneumorrhagia, n (%)	0 (0.00)	1 (8.33)	2 (3.92)	1.311	0.471*
Shock, n (%)	1 (7.14)	2 (16.67)	5 (9.80)	0.633	0.729
DIC^e^, n (%)	1 (7.14)	1 (8.33)	5 (9.80)	0.108	0.947
Total antibiotic duration, mean ± SD, days	9.57 ± 5.52	9.08 ± 4.36	12.08 ± 5.86	2.071	0.133
Length of stay, median (IQR), days	12.00 (6.00, 14.25)	9.00 (6.00, 14.25)	14.00 (7.00, 15.75)	2.043	0.137
Mortality, n (%)	4 (28.57)	0 (0.00)	2 (3.92)	7.328	0.023*

^a^ White blood cells, ^b^Procalcitonin, ^c^C-reactive protein

^d^Respiratory distress is manifested by rapid breathing, more than 60 breaths per minute, a rapid heart rate, chest wall retractions, expiratory grunting, nasal flaring, and blue discoloration of the skin during breathing efforts

^e^Disseminated intravascular coagulation

*Fisher’s exact test

### Comparison of GBS serotype distribution and clinical diagnosis in the two age groups

A total of 153 clinical diagnoses were established in 84 infants, including 86 cases belonging to the GBS-EOD group (pneumonia: 40, sepsis: 29, meningitis: 4, complications: 13) and 67 cases belonging to the GBS-LOD group (pneumonia: 16, sepsis: 25, meningitis: 21, complications: 5). The serotype distribution in these 84 infants on the basis of the clinical disease in the two age groups is presented in Table 4. The incidence of meningitis in the LOD group (*P* = 0.040) was statistically higher than that in the compared group, and the most predominant serotype was III [44.44% (20/45)], which induced meningitis in LOD.

**Table 4:** Clinical diagnosis and distribution of GBS serotypes in 84 infants in the two age groups

Group	Diagnosis	Ia	Ib	II	III	V	*χ*^2^ value	*P*
GBS EOD	Pneumonia	7	6	3	22	2	10.510	0.511*
GBS LOD	Pneumonia	3	4	0	9	0		
GBS EOD	Sepsis	6	2	2	18	1	9.028	0.671*
GBS LOD	Sepsis	2	4	0	19	0		
GBS EOD	Meningitis	1	2	0	1	0	19.433	0.040*
GBS LOD	Meningitis	0	1	0	20	0		
GBS EOD	Complications^a^	0	4	0	9	0	15.633	0.136*
GBS LOD	Complications^a^	2	0	0	3	0		
Total GBS EOD		13	13	5	56	3	9.095	0.059*
Total GBS LOD		8	10	0	45	0		

^a^ including pneumorrhagia, shock, and disseminated intravascular coagulation

* Fisher’s exact test

### Genetic diversity of serotypes Ia, Ib, II, III, and V

MLST analysis demonstrated the presence of 18 STs among 84 GBS isolates. ST17 was the most prevalent type [42.86% (36/84)], followed by ST23 [13.10% (11/84)], ST19 [10.71% (9/84)], ST12 [7.14% (6/84)], ST10 [4.76% (4/84)], ST27 [3.57% (3/84)], and ST24 and ST28 [both 2.38% (2/84)]. Other types, including ST1, ST8, ST88, ST268, ST485, ST651, ST652, ST862, ST890, ST1148, and the undetermined (UD) type, were also identified, but only one isolate was detected for each ST [all 1.19% (1/84)]. We performed MLST for the 14 isolates in serotype Ia and found that ST23 [78.57% (11/14)] predominated. Among the 12 isolates in serotype Ib, ST12 [50.00% (6/12)] was the most predominant, whereas ST17 was the most prevalent type [68.63% (35/51)] in the 51 isolates of serotype III. ST12 was specifically detected in serotype Ib, ST17/ST19 was specifically detected in serotype III, and ST23/ST24 was specifically detected in serotype Ia. ST23 was specifically detected in serotype Ia, ST12 was specifically detected in serotype Ib, and ST19/ST27 was specifically detected in serotype III. The percentage of ST17 in the GBS-LOD group was significantly higher than that in the GBS-EOD group [55.88% (19/34) vs 34.00% (17/50), *P* = 0.047]. The MLST results are shown in Tables 5 and 6.

**Table 5:** Multi-locus sequence typing (MLST) of the five serotypes among 84 GBS isolates

MLST	Ia	Ib	II	III	V	Total
ST10	0 (0)^a^	1 (1)	1 (0)	1 (0)	0 (0)	3 (1)
ST12	0 (0)	3 (3)	0 (0)	0 (0)	0 (0)	3 (3)
ST17	0 (0)	0 (0)	1 (0)	16 (19)	0 (0)	17 (19)
ST19	0 (0)	0 (0)	0 (0)	4 (4)	1 (0)	5 (4)
ST23	8 (3)	0 (0)	0 (0)	0 (0)	0 (0)	8 (3)
ST24	0 (0)	0 (0)	0 (0)	0 (0)	2 (0)	2 (0)
ST27	0 (0)	0 (0)	0 (0)	2 (1)	0 (0)	2 (1)
ST28	0 (0)	0 (0)	2 (0)	0 (0)	0 (0)	2 (0)
Others	ST1:1, ST88:1 (ST652:1)	ST268:1, ST862:1 (ST8:1, UD^b^:1)	0 (0)	ST485:1, ST651:1, ST890:1, ST1148:1 (0)	0 (0)	8 (3)

^a^GBS-EOD (GBS-LOD), ^b^UD: undetermined

**Table 6:** Comparison of GBS genetic distribution in the 84 GBS isolates between the two different age groups

MLST	GBS EOD (*n* = 50)	GBS LOD (*n* = 34)	*χ*^2^ value	*P*
ST10	3	1	0.015	0.901
ST12	3	3	0.004	0.951
ST17	17	19	3.957	0.047
ST19	5	4	0.000	1.000
ST23	8	3	0.394	0.530
ST24	2	0	–	0.512*
ST27	2	1	0.000	1.000
ST28	2	0	–	0.512*
Others	8	3	0.394	0.530

*Fisher’s exact test

## Discussion

There is a paucity of generalizable data on invasive GBS infections among infants in Asia. Additionally, there is a lack of data on the prevalence of invasive GBS infections among infants in China. Thus, we performed this study to assess serotype distribution and to obtain clinical and molecular microbiological information on invasive GBS disease among infants in southern mainland China that will help develop methods to prevent infant GBS infections.

In this study, we observed that GBS infections among infants were most frequently caused by serotype III (60.71%), followed by Ia (16.67%) and Ib (14.29%). The distribution of serotypes of GBS isolates in our study was similar to that reported for Beijing in 2012–2013 by Wang et al., which was as follows: III, 32.1%; Ia, 17.9%; and Ib, 16.1% [11]. The serotype distribution was also similar to that reported by Lo C-W et al. in Taiwan in 1998–2014, where serotype III caused 53.9% of infection episodes, followed by Ia with 17.0% and Ib with 10.4% [14]. A recent global review of 6500 invasive GBS isolates from infants showed that serotype III (61.5%) was predominant and 97% of cases were caused by serotypes Ia, Ib, II, III, and V [11], consistent with a previous study [15].

After presenting the serotype distribution according to the disease onset, we identified the predominance of serotype III in all age groups. Serotype Ia was the second most prevalent in early onset-GBS, whereas serotype Ib was the second most prevalent in late onset-GBS. Lo C-W et al. reported that serotype III induced approximately half of the infections, but serotype Ia was predominant in patients younger than 72 h [14]. A worldwide study revealed that serotype III caused nearly half (47%) of GBS-EOD cases and 73.0% of GBS-LOD cases [10]. Serotypes Ia, Ib, and V were frequently isolated from GBS-EOD (22.8, 8.0, and 10.6%, respectively) and GBS-LOD patients (14.2, 5.3, and 4.0%) [10]. Data from another global systematic review and meta-analysis [3] showed that 221 (37%) of 604 early-onset serotypes were type III compared with 347 (53%) of 653 late-onset serotypes and that 242 (40%) early-onset serotypes were type I in contrast with 196 (30%) late-onset serotypes. Thus, it was observed that disease-causing GBS serotypes in infants were similar in terms of prevalence across various regions, with some minimal variations depending on the geographic location, climate, and source of the bacterial isolates. However, there are only a few studies of GBS serotypes in Asia. Our study provides new data on the serotypes of invasive GBS isolates in a Chinese population.

We focused on differential demographics and the clinical presentations of the three most prevalent serotypes, Ia, Ib, and III. Although serotype III was more common, serotype Ia caused significantly higher rates of intrapartum temperature ≥ 37.5 °C, chorioamnionitis, and mortality. A Kaplan-Meier plot from this study revealed that patients with serotype III infections had a higher probability of survival than those with non-serotype III infections including serotype Ia infections, which was consistent with a previous study [14]. Apart from the lower age of patients with serotype Ia disease, hyper-virulence was considered to be more related to serotype Ia than to serotypes Ib and III. More research should be performed before we can understand the definite reasons for these findings. Basically, serotype III has high invasive potential and is the leader in causing invasive disease worldwide [3].

This study showed that respiratory distress occurred more frequently in patients with serotype Ia and Ib disease, whereas purulent meningitis was more related to serotype III. It has previously been reported that 86.2% of meningitis cases and 60.8% of sepsis cases were caused by serotype III GBS isolates in European countries and the United States [16]. Our data also demonstrated that meningitis was dominant in LOD cases compared to EOD cases, and that serotype III was the most prevalent serotype that induced meningitis in LOD cases, in agreement with a previous study [17]. Thus, serotype III GBS isolates were closely associated with purulent meningitis and serotype Ia tended to associate with pneumonia. This finding is probably due to the different virulence of GBS isolates with various serotypes [18]. The data revealed an increase in the number of cases of LOD meningitis caused by serotype III, consistent with a previous study [19]. Moreover, IAP generally has no effect on the incidence of LOD, and GBS can cause diseases in young infants older than three months [20].

There is limited data concerning MLST for GBS isolates from China. Our research assessed this aspect, and the results showed that ST17 was the most prevalent type (42.86%) among the 84 GBS strains, followed by ST23 (13.10%) and ST19 (10.71%). ST17 was the most common type (68.63%) in serotype III isolates. ST23 was detected specifically in serotype Ia. The percentage of ST17 in the LOD group was significantly higher than that in the EOD group. ST17 and ST19 were found almost exclusively in serotype III, which was in accordance with another study [21]. In the data from Taiwan, ST23 and ST24 comprised 85% of serotype Ia [14]. The ST17 clone, which mainly belonged to serotype III, was considered to be hypervirulent and related to meningitis [14]. This explains why ST17 was more frequently found in LOD in our study. Invasive GBS disease in infants is especially correlated with serotypes III (represented mainly by ST19 in Asia and ST17 in Europe) and Ia (represented mainly by ST23 and ST24) [22, 23].

GBS disease is not well recognized or reported in China. However, many reports from China show that GBS is a major infectious cause of morbidity and mortality among infants in Chinese population [24, 25]. In addition, China plans to relax the current two-child limit, which will allow married couples to have more than two children. This relaxation of the policy will result in the birth of more children every year. Thus, to reduce mortality, universal screening for maternal GBS colonization and subsequent IAP should be performed in China. We suggest that a safe and efficacious maternal vaccine against the most common serotypes should be developed and applied because LOD cannot be prevented by IAP [26]. The serotyping results and ST distribution in our study are important for selecting future GBS vaccines in China.

Our study has some limitations. These include the retrospective nature of the study, low number of GBS isolates obtained, and lack of data from northern China, which may have led to bias in the results.

## Conclusions

In summary, according to our epidemiological investigation of GBS, serotype III is the most common serotype and ST17 is the dominant genotype in southern mainland China. We identified some clinical correlations between various serotypes and the associated diseases. Maternal vaccination provides an alternative strategy, and our data suggest that a pentavalent conjugate vaccine (including Ia/Ib/II/ III/V) will cover nearly all disease-inducing GBS serotypes among young infants in China.

## Data Availability

The datasets generated and analyzed during the current study are available from the corresponding author on reasonable request.

## Abbreviations

GBS: Group B *streptococcus*
CPS: Capsular polysaccharide
EOD: Early-onset disease
LOD: Late-onset disease
MLST: Multi-locus sequence typing
